# Repair of damaged supraglottic airway devices: A novel method

**DOI:** 10.1186/1757-7241-18-33

**Published:** 2010-06-17

**Authors:** Manpreet Singh, Ritu Bharti, Dheeraj Kapoor

**Affiliations:** 1 Department Of Anaesthesiology and Intensive Care, Govt. Medical College & Hospital, Sector 32, Chandigarh, India

## Abstract

Damage of laryngeal mask airway and other supraglottic airway devices has always been a matter of concern. Although manufacturer recommends maximum 40 uses of LMA (and its congeners) but damage before 40 uses needs to be evaluated. We hereby, describe a novel method of repair of supraglottic devices when damage occurs at mask inflation line or pilot balloon valve assembly.

## Introduction

Various supraglottic airway devices like laryngeal mask airway (LMA), Proseal LMA, laryngeal tube etc. are novel innovative devices for upper airway management.Laryngeal mask airway and its variants are most often used in elective surgical procedures, emergency difficult airway management and in fields or camp surgeries. The currently manufactured model is made of silicone rubber and needs special care for its longitivity [1]. The device is used multiple times as supraglottic devices are mainstay of airway management now a days. Tracking the number of uses is very essential to prevent overuse of reusable LMA airways. Continued use of LMA airways beyond 40 uses increases the probability of device malfunctions for example fractured airway tubes, cuff tearing etc [2]. Even though manufacturers recommend 40 maximum uses of silicon LMA but damage of devices is not guaranteed even before 40 uses. The damages can also occur in emergency situations like cardiac arrest or in unanticipated difficult airway.Moreover, the financial constrains in developing countries does not allow its discard frequently and hence LMA and its variants are used repeatedly for more than 40 uses.

Biting of airway tube or LMA damage often occurs at cuff portion or airway tube shaft. The junction where airway tube is attached to the cuff may break while inserting laryngeal cuff in oral cavity and this may cause an irreparable loss of the equipment. The mask inflation line or valve assembly of supraglottic airway devices like LMA (and its congeners ) etc. can also damage while handling, washing or cleaning of expensive equipment. We hereby, recommend novel indigenous method of securing various supraglottic airways when damaged at mask inflation line or valve assembly.

### Apparatus

**Figure 1:**
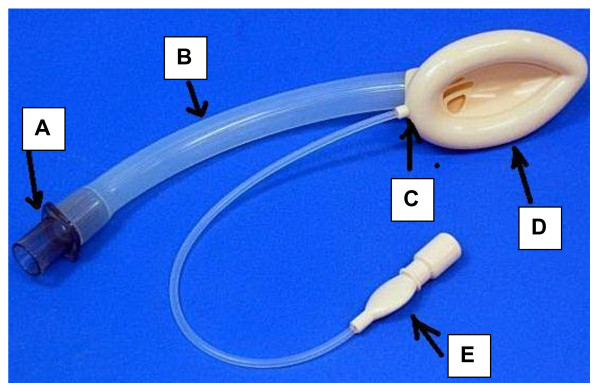
**Shows Laryngeal Mask Airway Classic**. A: Connector; B: Airway tube; C: Junction where airway tube is attached; D: Cuff; E: Pilot balloon assembly.

LMA and its variants contain mainly 4 parts- cuff, connector, airway tube and mask inflation line (with pilot balloon (Fig 1).

The mask inflation line consists of 3 parts -

1. Junction where line attaches to cuff

2. Inflation pilot balloon

3. Valve

Damage of pilot balloon, mask inflation line or valve assembly by either bitten by patient or during cleaning of equipment leads to loss of costly equipments permanently. This damage makes the equipment 'NOT USEFUL' and it is discarded immediately from the inventory. Moreover the damage can occur even after insertion or in the surgeries conducted at fields or camps. We hereby, have repaired this damage of costly SADs by affixing a threeway stopcock and a closed leur access split septum port (BD Q-Syte™) to the mask inflation line.

### Steps for repairing supraglottic device

**Figure 2:**
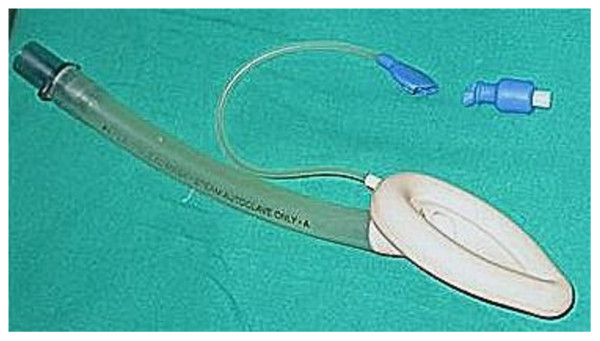
**Damaged LMA pilot balloon**.

**Figure 3:**
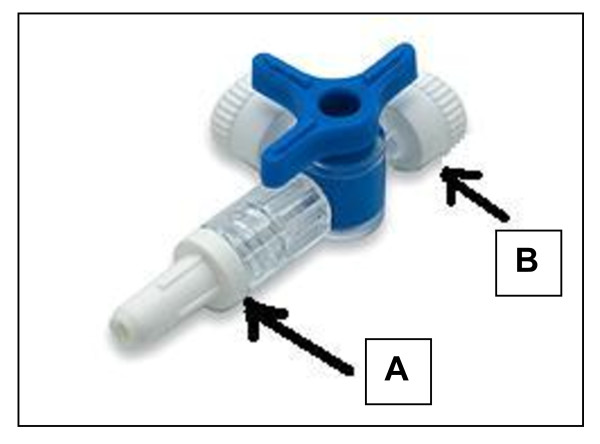
**Threeway stopcock**.

**Figure 4:**
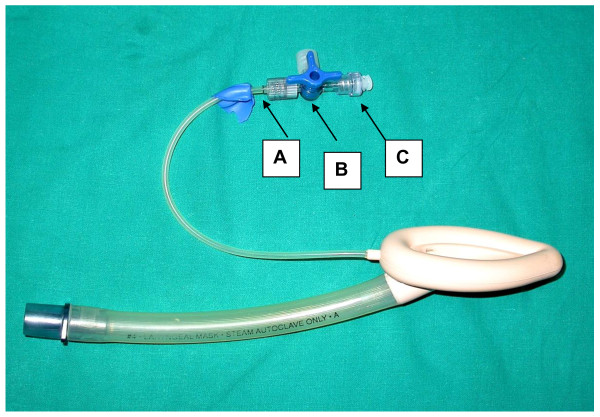
**Repaired LMA**. A: Teared end; B: Threeway stopcock; C: BD Q Syte connector.

The steps of repairing the supraglottic devices (LMA or its congeners) are as follows:

1. Expose and clear the damaged part of pilot balloon assembly (Fig. 2)

2. Attach mask inflation line (with medical adhesive) to 'Threeway Stopcock and closed leur access split septum port' assembly by inserting inflation line (teared end) inside End A of Threeway stopcock)(Fig. 3).

3. End B of Threeway stopcock is attached to closed leur access split septum port

4. Completely repaired LMA assembly is ready (Fig. 4).

The stopcock with luer lock is opened while inflating the pilot balloon with cuff inflator. As soon as desired inflation is completed luer lock is rotated and there is disconnection between inflation line and cuff inflator.

## Discussion

Supraglottic devices are the main stay in airway management in modern era. Since designing of LMA by Dr Archie Brain (UK) in 1981, it has been successfully used as a ventilatory device in both predicted and unpredicted difficult airway. In certain situations, such as cardiac arrest, there is no time to predict and/or act on the prediction of a difficult airway and supraglottic airway devices act as rescue airway management devices [3]. The other supraglottic devices like LMA (and its congeners), Laryngeal tube, laryngeal tube suction devices, etc have been successfully introduced from time to time in anaesthetic practice. In developing countries like India, cost has always been a major limiting factor for use of these devices. Moreover, the training of use of these expansive equipments is also essentially required.

During training period of residents, the probability of tearing of mask inflation line of any supraglottic devices is very high and consequently it leads to irrepairable loss of equipment. Further, the mask inflation line may get damaged during extubation phase and this can occur most often in patients with surgeries in psychiatric patients. In situations like cardiac arrest or unanticipated difficult airway if SAD is damaged from inflation line or valve assembly then it may create panic in anaesthesiologist's mind.

The present supraglottic equipment repairing technique is a novel indigenous method and can be useful for anaesthesiologists working in developing countries and working in fields/camps. This repairing can also be utilized in cases of patients with trauma and in emergency airway management when LMA or such expansive equipments may damage. This indigenous method can be applicable to expansive equipments like LMA proseal, LMA C-trach, Laryngeal Tube, Laryngeal Tube Suction, ILMA, ETT of ILMA etc. and the cost-effectiveness of equipment can be maintained by this simple, cheap and easier repair technique.
